# Anti-Inflammatory Effects of Artocarpin-Loaded Chitosan Microparticles on Inflammatory Chondrocytes

**DOI:** 10.3390/molecules31173129

**Published:** 2026-09-07

**Authors:** Piyapan Manklinniam, Ratsada Praphasawat, Arthid Thim-Uam, Grissana Pook-In, Jarupa Viyoch, Atchariya Yosboonruang

**Affiliations:** 1 Division of Microbiology and Parasitology, School of Medical Sciences, University of Phayao, Phayao 56000, Thailand; piyapan.up@gmail.com (P.M.); grissana.po@up.ac.th (G.P.-I.); 2 Department of Pathology, School of Medicine, University of Phayao, Phayao 56000, Thailand; ratsada.pr@up.ac.th; 3 Division of Biochemistry, School of Medical Sciences, University of Phayao, Phayao 56000, Thailand; arthid.th@up.ac.th; 4 Department of Pharmaceutical Technology, Faculty of Pharmaceutical Sciences, Naresuan University, Phitsanulok 65000, Thailand

**Keywords:** anti-inflammation, chondrocytes, artocarpin, chitosan microparticle, osteoarthritis

## Abstract

Artocarpin is a promising bioactive compound with anti-inflammatory potential; however, its limited aqueous solubility may restrict its direct biological application. Therefore, artocarpin-loaded chitosan microparticles (CSPs/AE) were prepared as a biocompatible carrier to improve artocarpin delivery. This study aimed to evaluate the cytocompatibility, antioxidant activity, and anti-inflammatory effects of CSPs/AE in lipopolysaccharide (LPS)-stimulated chondrocytes. CSPs/AE maintained high cell viability (>80% at 0.125 and 0.5 mg/mL) and showed significantly lower cytotoxicity than free artocarpin. Morphological and DAPI analyses confirmed preserved cellular and nuclear integrity without marked apoptosis. CSPs/AE at 0.25 mg/mL reduced LPS-induced intracellular ROS accumulation by approximately 79% relative to the LPS group. Western blot results demonstrated inhibition of LPS-induced NF-κB phosphorylation, with CSPs/AE at 0.5 mg/mL reducing p-NF-κB expression to approximately 0.66-fold of that observed in the LPS group. CSPs/AE also partially restored aggrecan and collagen II expression without promoting a fibrocartilage, as indicated by unchanged collagen I. These findings indicate that CSPs/AE attenuate oxidative stress and inflammatory signaling while preserving cartilage matrix components, highlighting their potential for cartilage-protective applications.

## 1. Introduction

Osteoarthritis (OA) is an age-associated degenerative joint disorder characterized by cartilage deterioration, structural bone changes, proteoglycan and type II collagen loss, and extracellular matrix (ECM) degradation, with pro-inflammatory cytokines, including interleukin-1 beta (IL-1β), tumor necrosis factor-alpha (TNF-α), and IL-6, playing key roles in promoting cartilage destruction, pain, impaired joint mobility, and disease progression [1,2,3]. Inflammatory stimuli, including LPS and pro-inflammatory cytokines such as IL-1β and TNF-α, can activate chondrocytes and upregulate matrix metalloproteinases (MMPs), particularly MMP-1, MMP-3, and MMP-13, leading to the breakdown of type II collagen and aggrecan, which are essential components of the cartilage extracellular matrix [4,5]. Current clinical management of osteoarthritis primarily focuses on alleviating inflammation and improving joint function. Although conventional osteoarthritis treatments, including intra-articular glucocorticoids, non-steroidal anti-inflammatory drugs (NSAIDs), and sodium hyaluronate injections, can alleviate symptoms, their ability to provide sustained cartilage protection remains limited [6,7]. Natural polymers have attracted increasing attention as drug-delivery materials because of their biocompatibility, biodegradability, and capacity to provide controlled and sustained release of bioactive compounds. Among these polymers, chitosan is particularly suitable for cartilage-related applications owing to its favorable biological properties and ability to form particulate delivery systems [8].

Artocarpin, a prenylated flavonoid, has been widely recognized for its potent anti-inflammatory activity, primarily through the suppression of key pro-inflammatory mediators. In inflammatory responses, excessive cytokine production—particularly TNF-α—is closely associated with the induction of cyclooxygenase-2 (COX-2), leading to amplification of inflammatory signaling [9]. Artocarpin has been reported to attenuate the expression of central cytokines such as IL-1β [10] and IL-6 [11], which play pivotal roles in chronic inflammation. In addition, artocarpin suppresses reactive oxygen species (ROS) and nitrite generation and interferes with apoptosis-related signaling pathways, including TNF-α activation and secretion [12]. The therapeutic application of artocarpin remains limited by its poor aqueous solubility and low bioavailability, which may restrict its effective delivery and biological activity. To overcome these limitations, artocarpin-loaded chitosan particles were previously developed as a biocompatible delivery system and were shown to exhibit controlled release behavior and anti-inflammatory activity in lipopolysaccharide (LPS)-stimulated RAW 264.7 macrophages [13]. These findings demonstrated the potential of chitosan-based particles to enhance the delivery and biological performance of artocarpin. However, their effects on articular chondrocytes, particularly under osteoarthritis-related inflammatory and oxidative stress conditions, have not yet been fully investigated. Moreover, their ability to modulate inflammatory signaling and preserve cartilage extracellular matrix components remains unclear. Therefore, the present study aimed to evaluate the cytocompatibility, antioxidant, anti-inflammatory, and chondroprotective effects of artocarpin-loaded chitosan microparticles (CSPs/AE) in LPS-stimulated chondrocytes.

Specifically, this study aimed to examine their inhibitory effects on pro-inflammatory cytokine production in LPS-stimulated human chondrocytes, as well as to evaluate the expression of cartilage-degrading enzymes, including MMPs, in LPS-challenged chondrocytes. In addition, the effects of the test compounds on extracellular matrix homeostasis were assessed by determining Type I and Type II collagen production. The study further explored intracellular oxidative stress by analyzing reactive oxygen species (ROS) generation in LPS-stimulated chondrocytes. Finally, cell death-related responses induced by LPS and modulated by the test compounds were evaluated using Western blot analysis.

## 2. Results and Discussion

Previous studies have explored various formulation strategies for artocarpin and artocarpin-containing Artocarpus extracts, including microparticles, nanoemulsions, nanoemulgels, and chitosan-based systems [14,15,16]. Artocarpin has also demonstrated antioxidant and anti-inflammatory effects [9]. However, these previous formulation studies have largely focused on topical, dermatological, or wound-healing applications. Building on our previous development of artocarpin-loaded chitosan particles, the present study evaluates their biological effects in LPS-stimulated human chondrocytes, with emphasis on oxidative stress, inflammation-associated responses, cartilage degradation, extracellular matrix homeostasis, and cell death-related signaling.

### 2.1. Cell Viability

**Figure 1:**
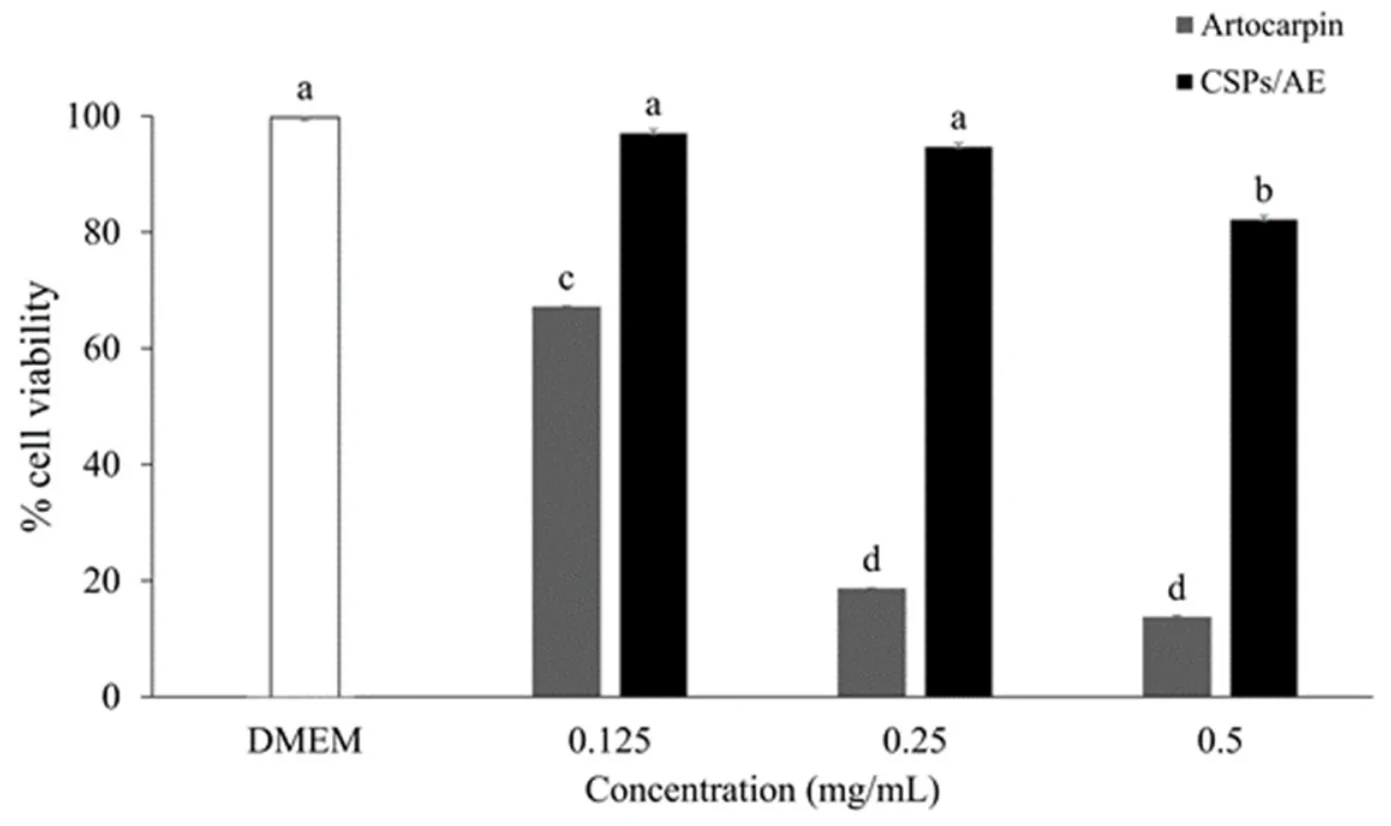
The chondrocyte cells viability of artocarpin and chitosan-loaded artocarpin (CSPs/AE) at different concentrations. Data are expressed as mean ± SD (*n* = 3). Different letters indicate significant differences among all groups according to one-way ANOVA followed by Tukey’s post hoc test (*p* < 0.05).

The cell viability of artocarpin and artocarpin-loaded chitosan particles (CSPs/AE) toward chondrocytes was evaluated using the MTT assay following 24 h exposure at concentrations ranging from 0.125 to 0.5 mg/mL. As shown in Figure 1, treatment with free artocarpin resulted in a significant reduction in cell viability in a dose-dependent manner. At concentrations of 0.25 and 0.5 mg/mL, cell viability markedly decreased, indicating potential cytotoxic effects of the free compound at higher doses. In contrast, CSPs/AE maintained significantly higher cell viability across all tested concentrations. At 0.125 and 0.25 mg/mL, cell viability remained above 90%, while even at 0.5 mg/mL, CSPs/AE preserved substantially higher viability compared with artocarpin alone. These findings demonstrate that encapsulation of artocarpin within the chitosan matrix effectively enhances cytocompatibility and reduces dose-related cytotoxicity.

**Figure 2:**
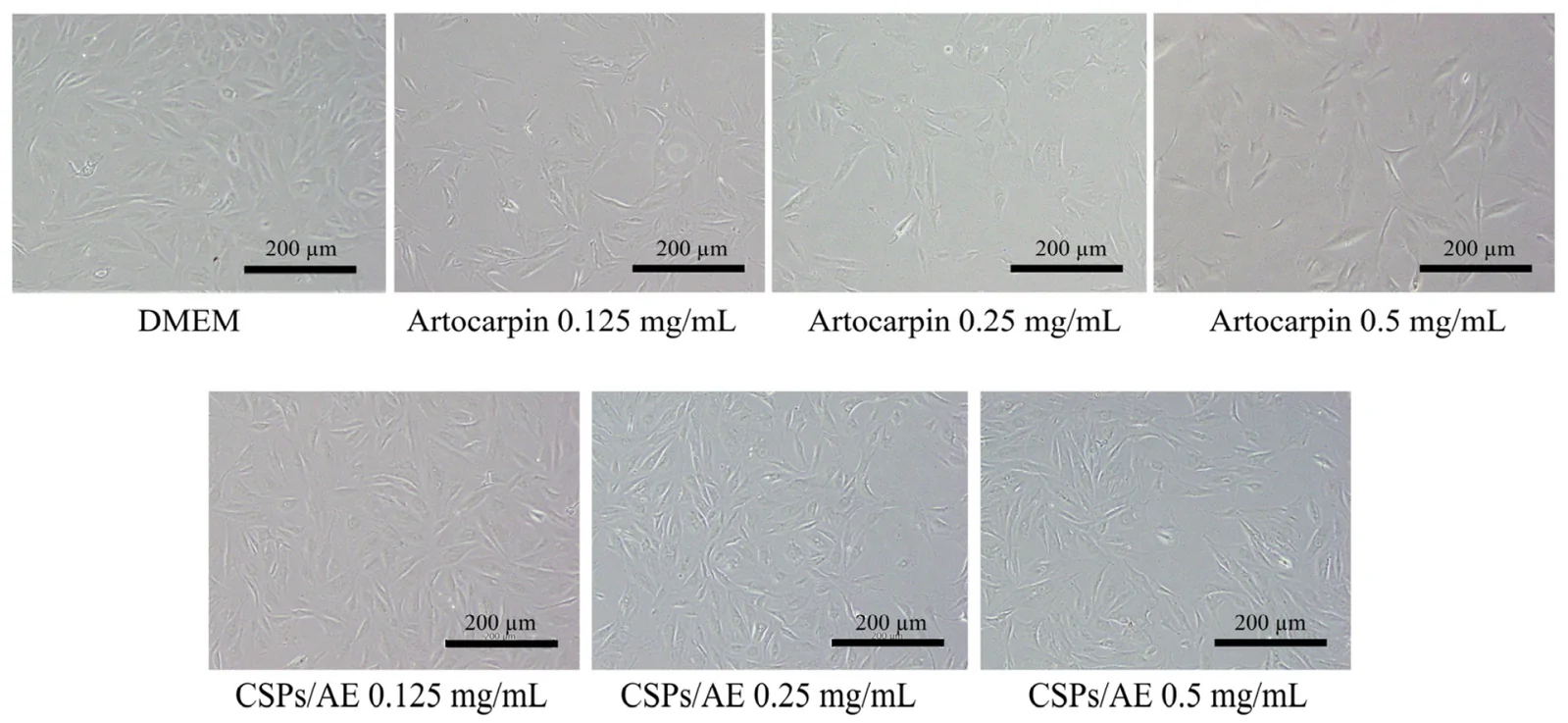
Morphology of chondrocytes after treatment with artocarpin and chitosan-loaded artocarpin (CSPs/AE) at different concentrations for 24 h. Cellular morphology was observed under an inverted microscope.

Morphological observations further supported the cell viability results (Figure 2). Untreated control cells (DMEM) exhibited the typical morphology of cultured chondrocytes with a relatively uniform distribution. Chondrocytes treated with CSPs/AE at 0.125 and 0.25 mg/mL showed morphology and cell density generally comparable to those of the untreated control. At 0.5 mg/mL, the cells also remained attached, with no apparent morphological deterioration or extensive cell detachment. Overall, no marked concentration-dependent changes in cellular morphology were observed within the tested concentration range, consistent with the cell viability results.

Although no significant reduction in cell viability was observed within the tested concentration range, dose-dependent cytotoxic responses are commonly reported for nanoparticle-based systems at higher exposures [17,18]. Elevated particle concentrations may promote aggregation or excessive membrane interactions, leading to cellular stress and reduced viability. Nevertheless, the high level of viability maintained at lower concentrations in this study is consistent with previous reports demonstrating the favorable biocompatibility of chitosan-based carriers [17,18,19]. Chitosan is widely recognized as a non-toxic, biodegradable, and bioactive polymer that supports cell survival and proliferation, making it a suitable material for biomedical and drug delivery applications [17,19,20]. The enhanced cytocompatibility observed for CSPs/AE may therefore be attributed to the intrinsic properties of chitosan, which can mitigate cellular stress and enable controlled release of encapsulated compounds [13,18,21].

Importantly, encapsulation of artocarpin within the chitosan matrix did not adversely affect cell viability, indicating that incorporation of this bioactive flavonoid does not compromise particle safety. Although artocarpin exhibits potent anti-inflammatory and antioxidant activities, certain flavonoids may exert pro-oxidant effects at elevated concentrations, potentially affecting cell survival [22]. The improved viability observed with CSPs/AE suggests that chitosan encapsulation modulates the release and cellular interaction of artocarpin, thereby minimizing potential cytotoxic effects while preserving its biological activity.

### 2.2. Cell Apoptosis Assay

**Figure 3:**
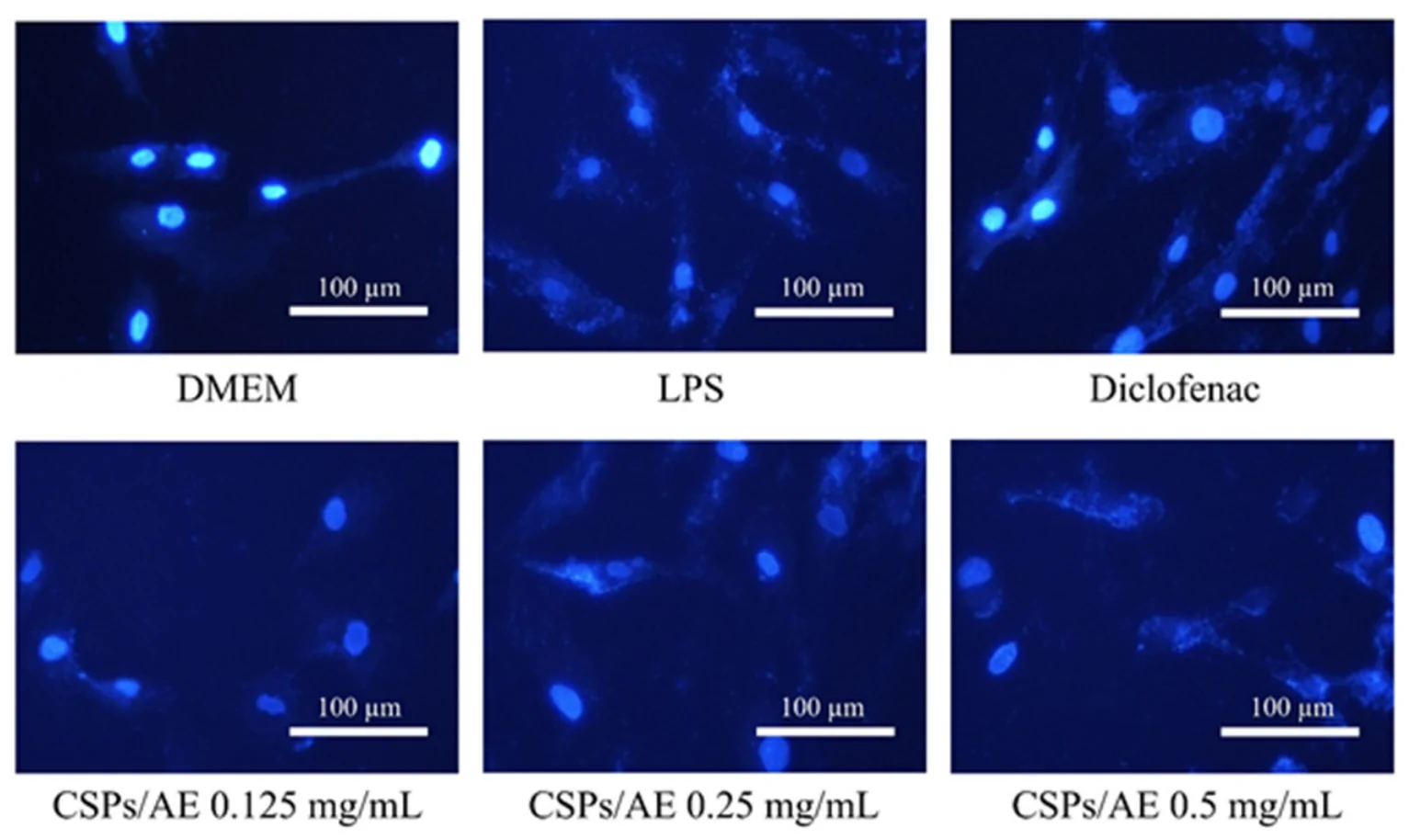
Evaluation of nuclear morphology in chondrocytes using DAPI staining. Representative fluorescence images of chondrocytes treated with DMEM, LPS, diclofenac, and CSPs/AE. DAPI-stained nuclei appear blue. LPS treatment resulted in alterations in nuclear morphology and DAPI staining, whereas diclofenac and CSPs/AE treatment showed relatively preserved nuclear morphology.

To evaluate the effects of CSPs/AE on nuclear morphology under inflammatory conditions, nuclear morphology was visualized using DAPI staining (Figure 3). In the DMEM control group, chondrocytes exhibited normal nuclear morphology, characterized by intact, round-to-oval nuclei with relatively homogeneous DAPI staining. LPS stimulation resulted in noticeable alterations in nuclear morphology and DAPI staining compared with the DMEM control, indicating LPS-induced cellular stress. In contrast, cells treated with CSPs/AE at 0.125 and 0.25 mg/mL showed relatively well-preserved nuclear morphology and more uniform DAPI staining compared with the LPS-treated group, indicating a relatively preserved nuclear morphology following treatment. At 0.5 mg/mL, some variation in nuclear morphology and staining intensity was observed; however, pronounced nuclear alterations were not readily apparent in the available images. Overall, these findings suggest that CSPs/AE, particularly at 0.125 and 0.25 mg/mL, may help preserve nuclear morphology and DAPI staining patterns in chondrocytes under LPS-induced inflammatory conditions.

The relatively preserved nuclear morphology following CSPs/AE treatment may be associated with the protective microenvironment provided by the chitosan matrix and the controlled release of artocarpin. As previously reported, the release profile of CSPs/AE was characterized by an initial burst release within the first 2–12 h, followed by a slower sustained release phase [13]. Initial burst release may facilitate immediate antioxidant and anti-inflammatory effects, thereby reducing early cellular stress under inflammatory conditions. Meanwhile, the sustained release phase helps maintain intracellular concentrations of artocarpin at non-toxic levels over time, which is essential for minimizing prolonged cellular stress and avoiding activation of apoptotic signaling pathways [23,24]. Controlled delivery systems are known to regulate intracellular exposure to bioactive compounds and help reduce stress-related cellular alterations.

Recent studies have demonstrated that chitosan-based carriers can mitigate oxidative stress and stabilize mitochondrial function, thereby supporting cellular homeostasis in various cell types [18]. The sustained release behavior of CSPs/AE likely contributes to maintaining intracellular homeostasis and reducing excessive cellular stress under inflammatory conditions.

Preservation of chondrocyte survival is essential for maintaining cartilage homeostasis, as excessive apoptosis is closely associated with cartilage degeneration and progression of osteoarthritis [25]. Inflammatory mediators and oxidative stress are key drivers of chondrocyte apoptosis through activation of mitochondrial and caspase-dependent pathways [26]. The ability of CSPs/AE to maintain nuclear morphology and relatively uniform DAPI staining patterns under inflammatory conditions therefore supports its potential as a biocompatible delivery system for cartilage-protective and anti-inflammatory applications.

### 2.3. ROS Assay

**Figure 4:**
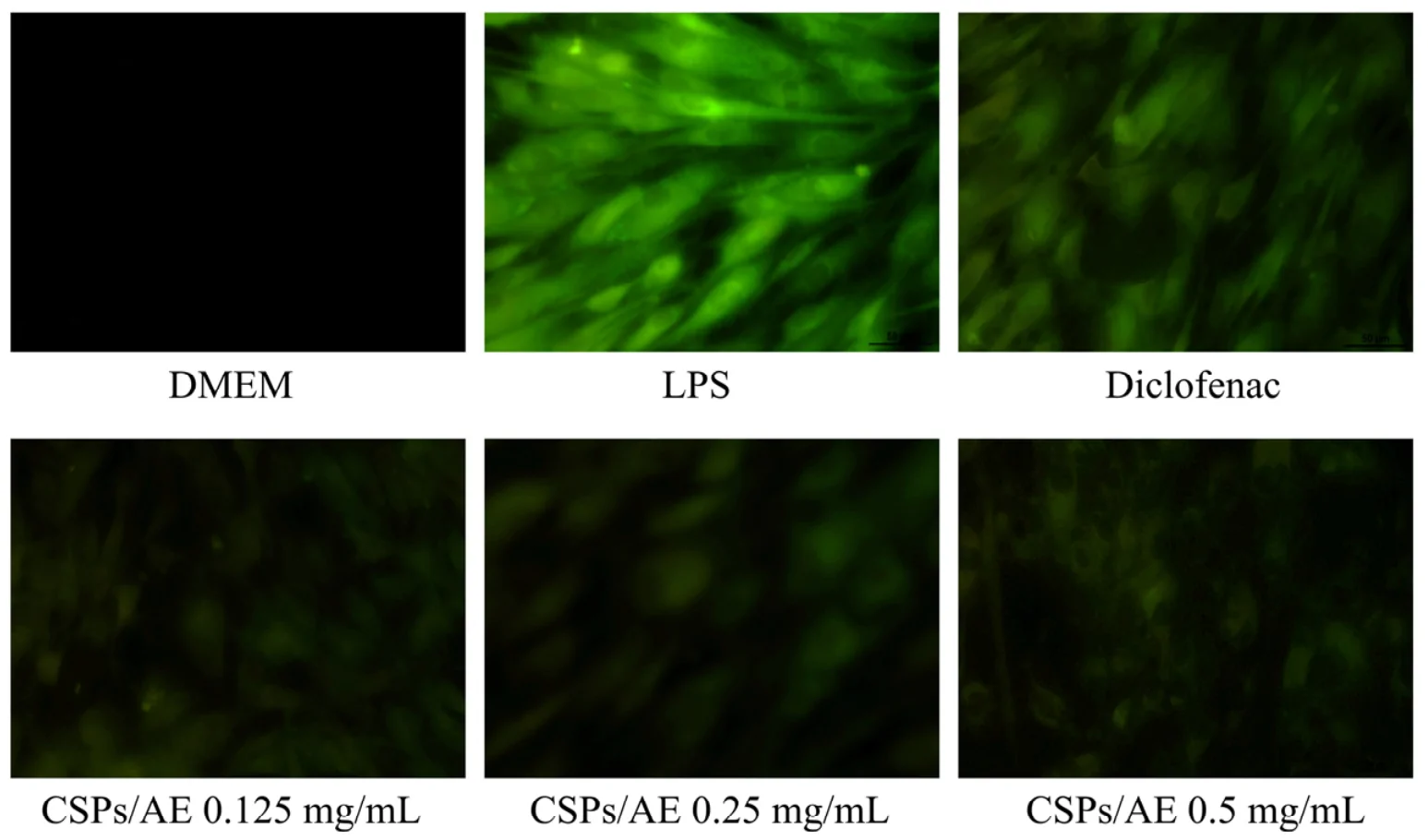
Determination of intracellular ROS generation in chondrocytes using the DCFH-DA assay. Fluorescence images of chondrocytes treated with DMEM, LPS, diclofenac, and CSPs/AE at concentrations of 0.125, 0.25, and 0.5 mg/mL for 24 h are shown. Intracellular ROS levels were detected using DCFH-DA fluorescence under identical exposure conditions.

ROS generation in chondrocytes was evaluated using the DCFH-DA assay following LPS stimulation and treatment with CSPs/AE. As shown in Figure 4, the control group showed very weak green fluorescence, indicating a low level of intracellular ROS. In contrast, LPS-stimulated cells showed a marked increase in fluorescence intensity, confirming that inflammatory stimulation significantly enhanced intracellular ROS generation. Treatment with diclofenac reduced green fluorescence compared with the LPS-treated group, although the fluorescence intensity observed in the representative image remained visually detectable.

**Figure 5:**
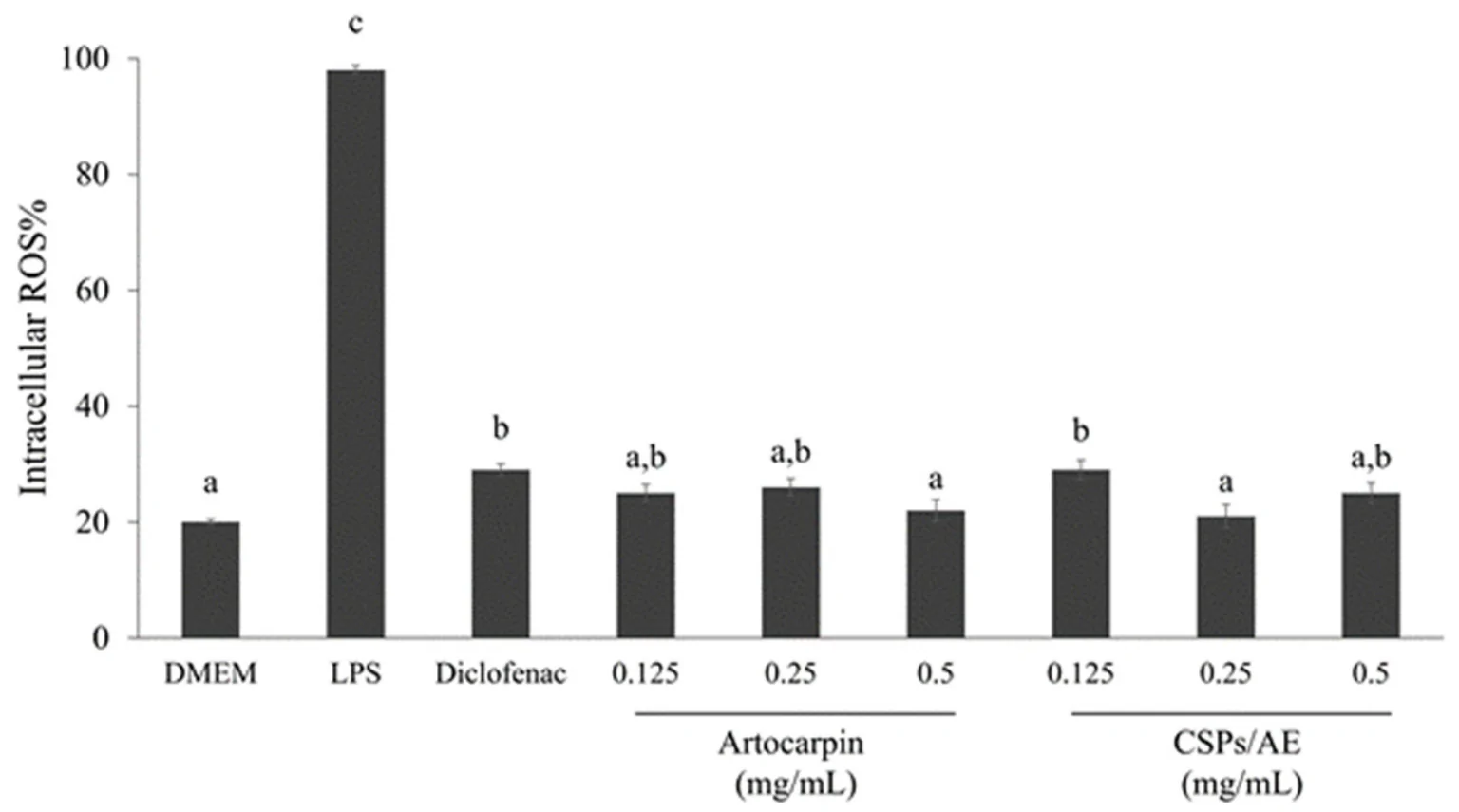
Fluorescence intensity of ROS in chondrocytes treated with free artocarpin and CSPs/AE at different concentrations. Chondrocytes treated with DMEM, LPS, and diclofenac were used as controls. Data are presented as mean ± SD (*n* = 3). Different letters indicate statistically significant differences among groups according to one-way ANOVA followed by Tukey’s post hoc test (*p* < 0.05).

Notably, treatment with CSPs/AE attenuated ROS generation in LPS-stimulated chondrocytes. Cells treated with CSPs/AE at concentrations of 0.125, 0.25, and 0.5 mg/mL exhibited lower green fluorescence intensity than the LPS-treated group. Because Figure 4 represents qualitative microscopic observations, differences in fluorescence intensity among the treatment groups were further evaluated using quantitative analysis. As shown in Figure 5, CSPs/AE significantly decreased ROS levels at all tested concentrations. Compared with the LPS-treated group, intracellular ROS levels were reduced by approximately 70%, 79%, and 75% following treatment with CSPs/AE at 0.125, 0.25, and 0.5 mg/mL, respectively. The greatest reduction was observed at 0.25 mg/mL, suggesting a concentration-specific rather than a strictly concentration-dependent response. Although CSPs/AE at 0.5 mg/mL retained substantial ROS-suppressive activity, this result should be interpreted together with the corresponding cell viability findings. Overall, CSPs/AE exhibited the most favorable ROS-inhibitory effect at cytocompatible concentrations, particularly 0.125–0.25 mg/mL.

Excessive ROS production is a key contributor to chondrocyte dysfunction and carti-lage degradation during inflammatory joint diseases. Elevated ROS levels can activate oxidative stress-mediated signaling pathways, leading to mitochondrial dysfunction, ex-tracellular matrix breakdown, and activation of pro-inflammatory mediators [27]. In this study, the marked increase in ROS following LPS stimulation confirms the establishment of an oxidative stress-driven inflammatory model in chondrocytes. The ability of CSPs/AE to significantly suppress ROS accumulation suggests that this formulation exerts a protective effect against inflammation-induced oxidative stress.

The observed reduction in ROS levels may be attributed to the combined properties of chitosan and artocarpin. Furthermore, incorporation of artocarpin into the chitosan ma-trix may enhance the overall redox-regulating capacity of the formulation by enabling sustained intracellular delivery and improved stability of the bioactive compound [21]. Such controlled release behavior can contribute to the attenuation of oxidative stress and modulation of redox-sensitive signaling pathways associated with inflammation and cellular damage [13]. Encapsulation of artocarpin within the chitosan matrix likely facili-tates sustained release and enhances its intracellular stability, thereby improving its ca-pacity to mitigate oxidative stress in chondrocytes.

### 2.4. Western Blot Assay

**Figure 6:**
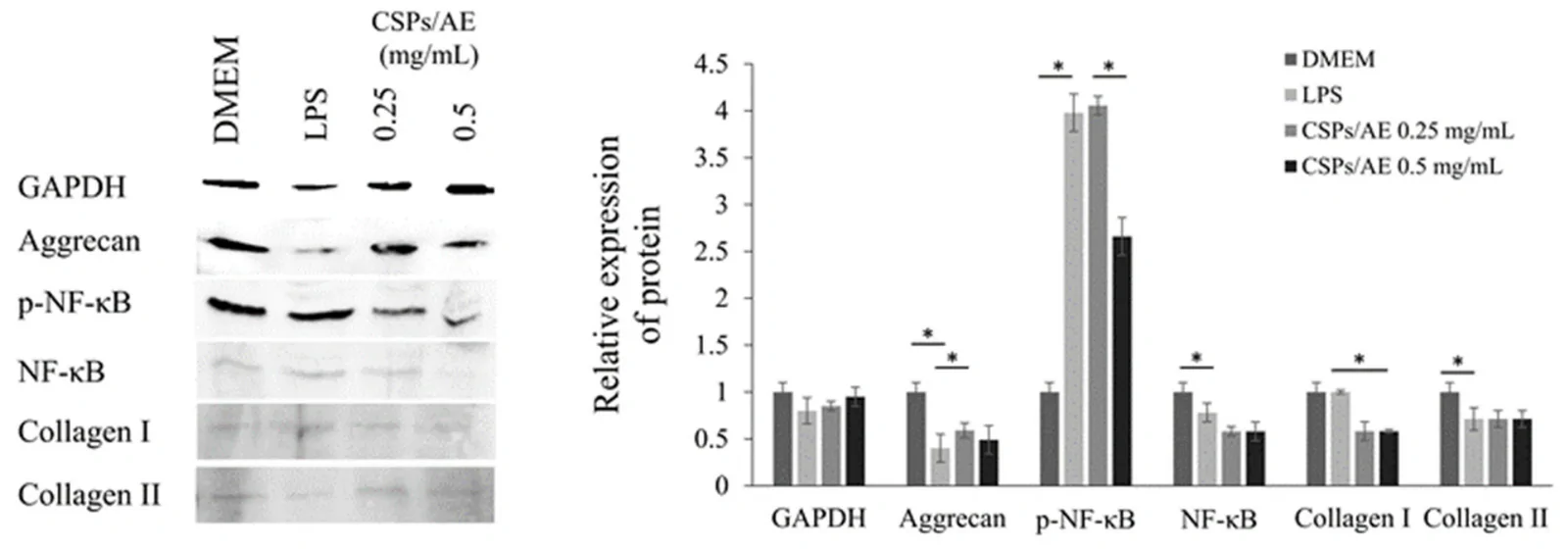
Western blot profiling of inflammatory and cartilage matrix-homeostatic proteins in LPS-stimulated chondrocytes following CSPs/AE treatment. Chondrocytes were treated with CSPs/AE at concentrations of 0.25 and 0.5 mg/mL. GAPDH was used as the loading control, * *p* < 0.05.

Western blot analysis was performed to evaluate the anti-inflammatory and chon-droprotective effects of CSPs/AE on LPS-stimulated chondrocytes (Figure 6 and in Supplementary Materials). As expected, LPS stimulation markedly increased the phosphorylation of NF-κB compared with the untreated control (DMEM), indicating activation of the NF-κB-mediated inflammatory signaling pathway. NF-κB is a central regulator of inflammation in osteoarthritis, controlling the transcription of pro-inflammatory cytokines, oxidative stress mediators, and matrix-degrading enzymes that contribute to cartilage breakdown and chondrocyte dysfunction [27,28]. Treatment with CSPs/AE at concentrations of 0.25 and 0.5 mg/mL significantly reduced the level of phosphorylated NF-κB in a dose-dependent manner, while total NF-κB expression remained relatively unchanged. This distinction is critical, as phosphorylated NF-κB (p-NF-κB) reflects the active form responsible for nuclear translocation and transcriptional regulation, whereas total NF-κB represents basal protein levels. The selective inhibition of NF-κB phosphorylation suggests that CSPs/AE suppresses inflammatory signaling without disrupting essential cellular regulatory mechanisms, which is considered a more favorable therapeutic strategy.

Regarding anti-inflammatory effects, CSPs/AE exerted a protective role on cartilage extracellular matrix components. LPS exposure markedly decreased the expression of key cartilage matrix proteins, including aggrecan and collagen II, consistent with inflammation-induced cartilage degradation. Notably, treatment with CSPs/AE restored the expression of aggrecan and collagen II compared with the LPS-treated group, indicating preservation of cartilage-specific matrix synthesis and maintenance of chondrocyte phenotype. In contrast, collagen I expression was not significantly increased following CSPs/AE treatment. This observation is particularly important, as collagen I is commonly associated with fibrocartilage formation and chondrocyte dedifferentiation, which are undesirable outcomes in cartilage repair and regeneration. The absence of collagen I upregulation indicates that CSPs/AE does not induce phenotypic transition away from native hyaline cartilage, thereby supporting its suitability for maintaining chondrocyte-specific function.

In addition to inflammatory signaling, ECM integrity was assessed by examining aggrecan and collagen II, key structural markers of functional articular cartilage. Aggrecan maintains cartilage hydration and compressive resistance, while collagen II provides tensile strength and mechanical stability [29]. Their reduction after LPS stimulation indicates inflammation-associated matrix degradation, a hallmark of osteoarthritis progression [27]. The ability of CSPs/AE to preserve these proteins suggests that the formulation may support matrix homeostasis and chondrocyte function while attenuating inflammatory responses.

The coordinated modulation of these molecular markers demonstrates that CSPs/AE exerts both anti-inflammatory and chondroprotective effects. Inhibition of NF-κB activation suggests suppression of upstream inflammatory signaling pathways that regulate downstream catabolic mediators responsible for ECM degradation. This is consistent with the restoration of aggrecan and collagen II expression, indicating a shift from a catabolic to an anabolic balance within the cartilage microenvironment. Furthermore, the lack of collagen I upregulation confirms preservation of the native chondrocyte phenotype and avoidance of dedifferentiation.

These findings are particularly significant, as oxidative stress and inflammation are key drivers of cartilage degeneration in osteoarthritis, promoting ECM breakdown and chondrocyte apoptosis [25,27]. The ability of CSPs/AE to simultaneously suppress inflammatory signaling and preserve ECM components highlights its therapeutic relevance in maintaining cartilage homeostasis.

### 2.5. Effects of CSPs/AE on Inflammation in Chondrocytes

**Figure 7:**
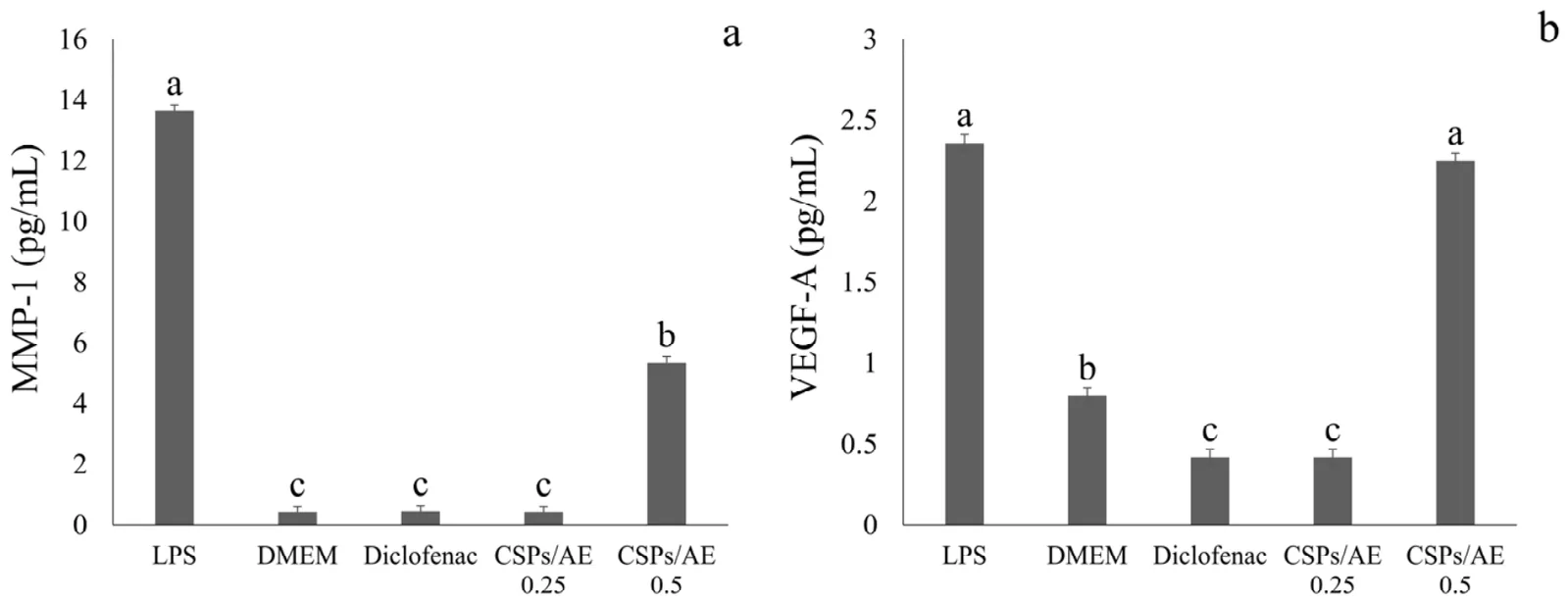
Effects of CSPs/AE on inflammatory and angiogenic mediators in LPS-stimulated chondrocytes. (**a**) MMP-1 levels and (**b**) VEGF-A levels were determined by ELISA following treatment with CSPs/AE at concentrations of 0.25 and 0.5 mg/mL. Different letters indicate statistically significant differences among groups according to one-way ANOVA followed by Tukey’s post hoc test (*p* < 0.05).

The effects of CSPs/AE on inflammatory and angiogenic mediators in LPS-stimulated chondrocytes were further evaluated by measuring MMP-1 and VEGF-A levels using ELISA (Figure 7). MMP-1 is a key matrix metalloproteinase involved in collagen degradation and is commonly associated with extracellular matrix breakdown during osteoarthritis progression [28]. In the present study, LPS stimulation increased MMP-1 levels compared with the DMEM control, indicating an enhanced catabolic response in chondrocytes. This finding is consistent with the involvement of MMP-1 in extracellular matrix degradation under inflammatory conditions [27].

In addition, previous studies have reported that artocarpin exhibits antioxidant and anti-inflammatory activities through multiple molecular mechanisms. It can directly scavenge ROS and activate the Nrf2-ARE pathway, thereby enhancing cellular antioxidant defense. In parallel, artocarpin has been reported to modulate key inflammatory signaling pathways, including NF-κB and MAPK, leading to reduced expression of pro-inflammatory mediators. Importantly, it also contributes to extracellular matrix preservation by inhibiting MMP while supporting collagen synthesis [29].

Treatment with CSPs/AE at 0.25 mg/mL further reduced MMP-1 levels to the lowest observed values, suggesting a marked inhibitory effect on matrix-degrading activity. This response occurred together with reduced NF-κB phosphorylation; however, a direct mechanistic relationship between NF-κB modulation and MMP-1 regulation cannot be established from the present data. At the higher concentration of 0.5 mg/mL, MMP-1 levels were partially increased compared with the 0.25 mg/mL group, but remained lower than those of the LPS-stimulated group. This finding suggests a dose-specific, rather than strictly concentration-dependent, response, with the strongest anti-catabolic effect observed at 0.25 mg/mL. Therefore, the response at 0.5 mg/mL should be interpreted with caution, as higher concentrations may be associated with altered cellular responses or reduced cytocompatibility.

VEGF-A, a key regulator of angiogenesis and cellular survival, exhibited a distinct concentration-specific response in this study. In articular cartilage, VEGF-A plays a complex role, being involved not only in angiogenesis but also in chondrocyte survival, metabolic activity, and tissue remodeling under both physiological and pathological conditions [6]. In the present study, LPS stimulation increased VEGF-A levels compared with the DMEM control, indicating that inflammatory stimulation altered VEGF-A-associated responses in chondrocytes. Treatment with CSPs/AE modulated VEGF-A levels in a concentration-specific manner. Notably, CSPs/AE at 0.25 mg/mL showed the greatest reduction in VEGF-A levels compared with the LPS-treated group, while maintaining acceptable chondrocyte viability and reducing intracellular ROS. However, given the complex biological functions of VEGF-A, these changes should not be interpreted as direct evidence of antiangiogenic activity. The biological significance of VEGF-A modulation by CSPs/AE therefore remains to be further clarified.

In contrast, CSPs/AE at 0.5 mg/mL restored VEGF-A expression to near control levels, indicating a possible role in supporting cellular survival and tissue repair. VEGF-A has been reported to contribute to cell survival signaling and adaptive responses under stress conditions, including enhancement of nutrient supply and modulation of cellular metabolism [7]. Therefore, the restoration of VEGF-A at higher concentrations may reflect improved cellular homeostasis rather than a purely pro-angiogenic effect.

The differential regulation of MMP-1 and VEGF-A highlights the multifunctional role of CSPs/AE in modulating both catabolic and adaptive responses in chondrocytes. The strong suppression of MMP-1 suggests inhibition of ECM degradation, while the restora-tion of VEGF-A at higher concentrations may support cellular viability and microenvironmental adaptation. These effects may be attributed to the combined antioxidant and anti-inflammatory properties of artocarpin, together with the controlled release behavior of the chitosan-based delivery system [9,11,30]. Collectively, the MTT, ROS, MMP-1, and VEGF-A results suggest that CSPs/AE modulated cellular responses in chondrocytes under LPS-induced inflammatory conditions. Among the tested concentrations, 0.25 mg/mL appeared to be the most suitable concentration, as it maintained cell viability and reduced ROS, MMP-1, and VEGF-A levels. In contrast, although 0.5 mg/mL still showed a protective effect, VEGF-A expression increased toward the control level. This finding suggests that the effect of CSPs/AE was not strictly dose-dependent, but differed according to the concentration used.

The favorable cytocompatibility profile of CSPs/AE supports its potential for further investigation as a delivery system for cartilage-related applications, particularly in osteoarthritis. Chitosan-based carriers have been extensively explored for their biodegradability, biocompatibility, and ability to enhance drug stability and sustained release, thereby improving cellular responses in biomedical applications [19]. In the present study, CSPs/AE demonstrated promising biocompatibility while maintaining chondrocyte viability, which is essential for preserving extracellular matrix integrity and preventing cartilage degeneration. These findings therefore suggest that CSPs/AE may represent a promising biocompatible delivery platform for artocarpin with potential cartilage-protective and inflammation-modulating effects. From a future clinical perspective, CSPs/AE may also possess potential immunomodulatory properties in addition to their direct chondroprotective effects. In the present study, CSPs/AE attenuated oxidative stress and NF-κB activation and reduced the levels of MMP-1 and VEGF-A in LPS-stimulated chondrocytes. Moreover, our previous study demonstrated that CSPs/AE reduced nitric oxide production in LPS-activated RAW 264.7 macrophages, suggesting that this formulation may influence macrophage-mediated inflammatory responses. Collectively, these findings indicate that CSPs/AE may contribute to modulation of the inflammatory microenvironment associated with osteoarthritis. However, because immune-cell responses were not directly evaluated in the present study, the immunomodulatory effects of CSPs/AE remain to be confirmed.

Nevertheless, further investigations are required to evaluate long-term cytocompatibility, in vivo safety, and interactions with other relevant cell types involved in joint pathology, including synoviocytes, fibroblasts, and immune cells. Future studies should also investigate macrophage polarization and the regulation of pro- and anti-inflammatory cytokines, including TNF-α, IL-1β, IL-6, and IL-10, to further clarify the immunomodulatory potential of CSPs/AE. In addition, elucidation of the molecular mechanisms underlying the maintained cellular viability, particularly those associated with oxidative stress regulation, inflammatory cytokine modulation, and apoptosis-related signaling pathways, would provide deeper insight into the biological effects of CSPs/AE and support the rational development of future chitosan-based nanocarrier systems [25,26].

## 3. Materials and Methods

Low-molecular-weight chitosan derived from crab shells, with a degree of deacetylation ≥ 75% and a viscosity ≤ 15 mPa·s, was purchased from Maruzen Chemicals Co., Ltd. (Tokyo, Japan). Sodium tripolyphosphate (TPP) was obtained from Thermo Fisher Scientific (Waltham, MA, USA). 2′,7′-dichlorodihydrofluorescein diacetate (DCFH-DA) was purchased from MySkinRecipes Co., Ltd., Bangkok, Thailand. Dulbecco’s Modified Eagle Medium (DMEM), fetal bovine serum (FBS), trypsin-EDTA, and penicillin/streptomycin were purchased from Gibco Invitrogen™ (Grand Island, NY, USA). MTT (3-(4,5-Dimethylthiazol-2-yl)-2,5-Diphenyltetrazolium Bromide) was purchased from TCI (Tokyo, Japan). Radioimmunoprecipitation assay (RIPA) lysis buffer was obtained from HiMedia Laboratories (Mumbai, India). Antibodies were purchased from Gibco Invitrogen™ (Grand Island, NY, USA).

### 3.1. Fabrication of CSPs/AE

Chitosan microparticles were prepared by ionotropic gelation as previously described [13]. The artocarpin extract (AE) was obtained from the heartwood of *Artocarpus altilis*. Briefly, dried and powdered heartwood was macerated with diethyl ether at a solid-to-solvent ratio of 1:6 (*w*/*v*) at room temperature for two consecutive 2-day cycles, as previously described [20]. The extracts were filtered, pooled, and concentrated under reduced pressure. The crude extract was subsequently fractionated by silica gel column chromatography, and artocarpin-containing fractions were identified by TLC, pooled, and concentrated to obtain the artocarpin-enriched extract.

For microparticle preparation, chitosan (0.1% *w*/*v*) dissolved in 1.0% (*v*/*v*) acetic acid was crosslinked with TPP (0.5% *w*/*v*) at a chitosan volume ratio of 5:1 under magnetic stirring. AE was dissolved in 3% (*v*/*v*) aqueous propylene glycol and incorporated into the chitosan solution at a final concentration of 0.5 µg/mL, corresponding to an initial AE-to-chitosan mass ratio of 1:2000 (*w*/*w*), prior to ionotropic gelation. The resulting suspension was stirred overnight at room temperature and centrifuged at 10,000 rpm for 25 min at 25 °C. The collected microparticles were washed twice with deionized water and dried to obtain the final artocarpin-loaded chitosan microparticles (CSPs/AE).

Chitosan microparticles were prepared by ionotropic gelation as previously described [13]. Briefly, chitosan (0.1% *w*/*v*) in 1.0% acetic acid was crosslinked with TPP (0.5% *w*/*v*) at a chitosan:TPP volume ratio of 5:1 under magnetic stirring. Artocarpin extract, dissolved in 3% (*v*/*v*) aqueous propylene glycol, was incorporated into the chitosan solution prior to gelation. The resulting suspension was stirred overnight at room temperature, followed by centrifugation (10,000 rpm, 25 min, 25 °C) and washing twice with deionized water to obtain the final microparticles (CSPs/AE).

### 3.2. Cytotoxicity Investigation

#### 3.2.1. Cell Viability

For cell culture, Human primary chondrocyte cell line (HUM-iCell-s018) was acquired from iCell Bioscience Inc., Shanghai, China. The cells were cultured and maintained in DMEM high-glucose medium containing 5% FBS and 100 units/mL of penicillin–streptomycin. The cell cultures were incubated in a carbon dioxide incubator at 37 °C with 90% humidity and 5% CO_2_.

Cells were seeded in 96-well plates at a density of 1 × 10^4^ cells/well and allowed to attach for 18 h. Artocarpin, either in free form or loaded into chitosan-based particles (CSPs/AE), was prepared as a stock solution in DMSO and diluted with serum-free DMEM to final concentrations of 0.125, 0.25, and 0.5 mg/mL. The cells were then treated with the prepared formulations and incubated for 24 h. Following treatment, cells were washed twice with phosphate-buffered saline (PBS) and incubated with MTT [3-(4,5-dimethylthiazol-2-yl)-2,5-diphenyltetrazolium bromide] solution for 3 h to allow formazan formation. The resulting formazan crystals were dissolved in DMSO, and absorbance was measured at 570 nm using a microplate reader (ETY-96; Toyosokki Co., Ltd., Tokyo, Japan). Cell viability was expressed as a percentage relative to untreated control cells, which were defined as 100% viability. The viability of treated cells was determined by normalizing the absorbance values of the treatment groups to those of the untreated control group.

#### 3.2.2. Cell Apoptosis Assay

Chondrocytes were seeded in 6-well plates at a density of 5 × 10^5^ cells/well and incubated overnight to allow cell attachment. After cell attachment, the cells were pretreated with artocarpin or CSPs/AE at final concentrations of 0.125, 0.25, and 0.5 mg/mL for 24 h. The treatment medium was then removed, and the cells were gently washed with serum-free DMEM, followed by stimulation with LPS for 20 h to induce inflammatory stress. Untreated cells, vehicle-treated cells, and LPS-stimulated cells were used as the normal control, vehicle control, and inflammatory model control, respectively, while diclofenac was used as a reference anti-inflammatory drug. Following treatment, cells were fixed with 4% paraformaldehyde for 30 min and washed with PBS three times. Subsequently, the chondrocytes were stained with 1 μg/mL DAPI (4′,6-diamidino-2-phenylindole) for 5 min and then washed with PBS. The cellular morphology was observed under an inverted fluorescent microscope.

### 3.3. Anti-Inflammation Activity Investigation

#### 3.3.1. ROS Assay

Intracellular reactive oxygen species generation in chondrocytes was evaluated using the 2′,7′-dichlorodihydrofluorescein diacetate (DCFH-DA) assay, as previously described with modifications [7]. Cells were seeded onto 8-well chamber slides at a density of 2 × 10^4^ cells/well and incubated at 37 °C in a humidified atmosphere containing 5% CO_2_ for 18 h to allow cell attachment. After cell attachment, the culture medium was removed, and the cells were treated with artocarpin or CSPs/AE prepared in serum-free DMEM at final concentrations of 0.125, 0.25, and 0.5 mg/mL for 24 h. The treatment medium was then removed, and the cells were gently rinsed with serum-free DMEM. Subsequently, the cells were stimulated with LPS prepared in serum-free DMEM for 20 h. Untreated cells, vehicle-treated cells (DMSO), and LPS-stimulated cells were used as the normal control, vehicle control, and inflammatory model control, respectively. After LPS stimulation, the medium was removed, and the cells were washed with serum-free DMEM. The cells were then incubated with DCFH-DA (10 μM) prepared in serum-free DMEM for 20 min at 37 °C in the dark. After incubation, the cells were washed three times with serum-free DMEM to remove excess probe that had not entered the cells. Intracellular ROS levels were detected using the DCFH-DA fluorescent probe (excitation at 488 nm and emission at 525 nm) under identical exposure settings for all experimental groups. In parallel, quantitative fluorescence measurements were obtained using a fluorescence microplate reader (Cytation 5, BioTek Instruments, Winooski, VT, USA) at the same excitation and emission wavelengths.

#### 3.3.2. Western Blot Assay

Chondrocytes were seeded in 6-well plates as described above and incubated to allow cell attachment. The cells were pretreated with CSPs/AE at concentrations of 0.25 and 0.5 mg/mL for 24 h, followed by LPS stimulation for an additional 20 h to induce inflammatory responses. DMEM-treated cells and LPS-stimulated cells were used as the negative control and inflammatory model control, respectively. After 48 h of treatment, the chondrocytes were lysed using RIPA lysis buffer for subsequent protein analysis. The proteins were prepared according to the previous protocol [31]. Proteins were separated by SDS-PAGE and subsequently transferred onto nitrocellulose membranes. Then the membranes were incubated overnight at 4 °C with primary antibodies against GAPDH, collagen I, II, aggrecan, p-NF-κB, and NF-κB antibodies. After washing with Tris-buffered saline with Tween-20 (TBST), the membranes were incubated with appropriate horseradish peroxidase-conjugated secondary antibody against for 1 h at room temperature. Goat anti-rabbit IgG secondary antibody was used for collagen II, aggrecan, p-NF-κB, and NF-κB, whereas goat anti-mouse IgG secondary antibody was used for GAPDH and collagen I. Protein bands were then visualized using a chemiluminescent detection system with the iBright™ CL1500 Imaging System (Invitrogen, Carlsbad, CA, USA). GAPDH was used as an internal control.

#### 3.3.3. ELISA Assay

Chondrocytes were seeded in 6-well plates as described above and incubated to allow cell attachment. The cells were pretreated with CSPs/AE at concentrations of 0.25 and 0.5 mg/mL or diclofenac for 24 h, followed by LPS stimulation for an additional 24 h to induce inflammatory responses. DMEM-treated cells and LPS-stimulated cells were used as the negative control and inflammatory model control, respectively. After 48 h of treatment, the chondrocytes were lysed using RIPA lysis buffer for subsequent protein analysis. After the designated treatment period, the culture media were collected and centrifuged at 3000 rpm for 10 min to remove cell debris. The concentrations of matrix metalloproteinase-1 (MMP-1) and vascular endothelial growth factor-A (VEGF-A) in the clarified culture supernatants were determined using ELISA kits (PPX-06-MXAADYD; Thermo Fisher Scientific, Vienna, Austria; distributed by Gibthai Co., Ltd., Bangkok, Thailand) in accordance with the manufacturer’s protocol. Fluorescence signals were acquired using a Luminex^®^ 200™ System based on xMAP^®^ technology (Luminex Corporation, Austin, TX, USA). Cytokine concentrations were calculated based on standard curves and expressed as pg/mL.

### 3.4. Statistical Analysis

All experiments were performed in triplicate. Data are presented as mean ± standard deviation (SD). Statistical analysis was conducted using one-way analysis of variance (ANOVA) followed by Tukey’s post hoc test using IBM SPSS Statistics (version 30.0). Differences were considered statistically significant at a *p*-value less than 0.05.

## 4. Conclusions

Artocarpin-loaded chitosan microparticles (CSPs/AE) demonstrated good cytocompatibility and protective biological activity in LPS-stimulated chondrocytes. CSPs/AE maintained cell viability, preserved normal cellular morphology, and reduced apoptotic features, while attenuating intracellular ROS generation and suppressing NF-κB activation. In addition, CSPs/AE helped preserve key cartilage extracellular matrix components, including aggrecan and collagen II, without inducing collagen I expression, suggesting maintenance of the chondrocyte phenotype and ECM integrity. CSPs/AE also modulated inflammatory and adaptive mediators in a concentration-specific manner. Among the tested concentrations, 0.25 mg/mL showed the most favorable response, as it maintained cell viability while reducing ROS production, MMP-1 levels, and VEGF-A expression. At 0.5 mg/mL, CSPs/AE still showed an overall protective tendency; however, the restoration of VEGF-A toward the control level indicates that the biological response was not strictly dose-dependent. These effects may be related to the combined antioxidant and anti-inflammatory properties of artocarpin, together with the biphasic release behavior of the chitosan-based delivery system.

Collectively, CSPs/AE may serve as a promising and biocompatible platform for cartilage-protective and anti-inflammatory applications. However, further mechanistic studies and in vivo investigations are required to confirm their long-term safety, biological mechanisms, and therapeutic potential.

## Data Availability

The original contributions presented in this study are included in the article. Further inquiries can be directed to the corresponding authors.

## Supplementary Materials

The following supporting information can be downloaded at: https://www.mdpi.com/article/10.3390/molecules31173129/s1, Figure 6 raw data or supplementary WB revise V1.
